# Psychological and Social Factors Associated With Reporting Post‐COVID Symptoms Among German Healthcare Workers: A Cross‐Sectional Study

**DOI:** 10.1111/jocn.17608

**Published:** 2025-01-14

**Authors:** Valentin Schick, Marietta Lieb, Andrea Borho, Eva Morawa, Franziska Geiser, Petra Beschoner, Lucia Jerg‐Bretzke, Christian Albus, Susann Steudte‐Schmiedgen, Andreas M. Baranowski, Sabine Mogwitz, Yesim Erim

**Affiliations:** ^1^ Department of Psychosomatic Medicine and Psychotherapy Friedrich‐Alexander‐University Erlangen‐Nürnberg (FAU) Erlangen Germany; ^2^ Department of Psychosomatic Medicine and Psychotherapy University of Bonn, Medical Faculty and University Hospital Bonn Germany; ^3^ Department of Psychosomatic Medicine and Psychotherapy Ulm University Medical Center, University Ulm Ulm Germany; ^4^ Department of Psychosomatic Medicine and Psychotherapy, Christophsbad Hospital Göppingen Germany; ^5^ Department of Psychosomatics and Psychotherapy University of Cologne, Medical Faculty and University Hospital Cologne Germany; ^6^ Department of Psychotherapy and Psychosomatic Medicine Faculty of Medicine and University Hospital Carl Gustav Carus, TUD Dresden University of Technology Dresden Germany

**Keywords:** COVID‐19, HCW, mental health, post‐COVID, resources, SarS‐CoV‐2, socioeconomic status, working condition

## Abstract

**Background:**

Health care workers (HCW) with post‐COVID condition (PCC) are frequently reported to suffer from mental health impairment. Given HCW above‐average risk for mental health, research is necessary and risk factors need to be assessed.

**Aim:**

To compare mental health and health of German HCW with and without PCC and to identify associated psychological and social factors.

**Design:**

Cross‐sectional study.

**Method:**

Overall, 2816 German HCW participated (332 reported PCC). Measures were post‐COVID condition symptom sum score (PCSS), symptoms of depression (Patient Health Questionnaire‐2), anxiety (Generalised Anxiety Disorder‐2) and post‐traumatic stress disorder (PTSD) symptoms (Impact of Event Scale‐6), work–family conflict (Work–Family Conflict Scale), social support (ENRICHD Social Support Inventory), sense of coherence (Sense Of Coherence‐3 Scale) and working conditions. Group differences of HCW with and without PCC were investigated. Multiple linear regression analysis was performed for HCW with PCC. PCSS was the dependent variable. Independent variables were a.m. measures and age, gender, occupational group and migration background.

**Results:**

HCW with PCC exhibited higher values, with medium effect sizes, for symptoms of depression, anxiety and PTSD. Small effect sizes were observed for work–family conflict, social support, sense of coherence and working conditions. Higher PCSS scores were associated with higher depression, anxiety, PTSD and work–family conflict levels, lower social support and sense of coherence and migration background. Being a physician was associated with lower PCSS.

**Conclusion:**

Lower mental health, social factors and resources may play a role in reporting severe post‐COVID symptoms. Further research is necessary to investigate these interactions using the biopsychosocial theory.

**Implication for the Profession:**

This study can help to understand PCC in HCW to design adjusted treatments and protect HCW from PCC and minimise their risk of PCC.

**Reporting Method:**

This study complies with the Journal article reporting standards for quantitative research in psychology: The APA Publications and Communications Board task force report (Data S1).

**Public Contribution:**

Caregivers are the sample group.

**Summary**

- What does this paper contribute to the wider global clinical community?
  ○ This paper reports on post‐COVID in German HCW workers reporting PCC after the pandemic from April to June 2023. Even if PCC was self‐reported, HCW with PCC differentiated from HCW without PCC with overall worse health and mental health specifically.
  ○ Besides worse mental health, lower resources and socioeconomic status were associated with reporting higher PCSS values.

## 1 Introduction

Following an initial SARS‐CoV‐2 (COVID‐19) infection, people are known to frequently suffer from postacute sequelae of COVID‐19. The illness describes a condition that ‘occurs in individuals with a history of probable or confirmed SARS‐CoV‐2 infection, usually three months from the onset, with symptoms that last for at least two months and cannot be explained by an alternative diagnosis’ (Soriano et al. 2022). The prevalence rate of post‐COVID condition (PCC) varies across populations. A German‐based study of predominantly nonhospitalised cases showed a prevalence rate of 6.5% at 6–12 months postinfection (Peter et al. 2022). The World Health Organisation (WHO) estimated a higher PCC prevalence between 10% and 20% (Rajan et al. 2021). PCC encompasses multiple types of symptoms. While over 200 symptoms are reported for similar long‐COVID, many impact mental health (Davis et al. 2021). A systematic review of 18 studies highlights the following psychological symptoms as the most frequent 12 months after an initial COVID‐19 infection (Han et al. 2022): Fatigue/weakness, depression, anxiety, memory loss, concentration difficulties and insomnia. A cross‐sectional study with a Japanese‐Swedish sample showed a multiplied risk with odds ratios for developing mental disorders for people with PCC, with 2.96 (95% CI: 1.29–6.79) for depression, 3.43 (95% CI: 1.30–9.31) for anxiety and 2.44 (95% CI: 1.10–5.43) for traumatic stress symptoms (Matsumoto et al. 2022). A meta‐analysis suggests a prevalence of 22% for anxiety symptoms (95% CI: 15–29) and 23% (95% CI: 12–34) for depression (Tebeka et al. 2023). Commonly reported risk factors for post‐COVID/long‐COVID are female gender, age and severity of acute infection (Gaber, Ashish, and Unsworth 2021; Menges et al. 2021; Muller et al. 2023; Sudre et al. 2021). The pathogenesis of PCC appears to be complex, and interdisciplinary diagnostic approaches have been attempted, but to date, no causal treatments have been found, nor has the pathogenesis behind the illness been fully uncovered. COVID‐19 impacted socioeconomically weak groups more than socioeconomically strong groups (Morrow et al. 2024). To improve the understanding of the PCC mechanism, it is worth investigating social and occupational groups that PCC distinctively impacts.

## 2 Background

Here, a group strongly impacted by the COVID‐19 pandemic were health care workers (HCW). Large amounts of HCW were mentally burdened during COVID‐19: In 2020, 20.9% of German HCW were above the clinically significant cut‐off for PHQ‐2 and 19.1% for GAD‐2, measuring depression and anxiety respectively (Morawa et al. 2021). After episodes of COVID‐19, HCW suffered disproportionally from PCC. Between 2021 and 2022, the most affected professions to be unable to work due to PCC were 7 out of 10, all in health care (Meyer, Meinicke, and Schenkel 2023). In other parts of the world, HCW were also impacted by the pandemic. In Saudi Arabia, HCW reported depressive symptoms (60.9%), stress symptoms (56.3%) and anxiety symptoms (63.3%) during spring 2020 (Abuzied et al. 2021). UK HCW with long‐term COVID‐19 complications were reluctant to receive medical advice during the pandemic (Gaber, Ashish, and Unsworth 2021). UK HCW reported 39% fatigue, 40% shortness of breath, 49% sleep disturbance and 45% mood disorder after their initial COVID‐19 infection (Gaber, Ashish, and Unsworth 2021). The pre‐COVID‐19 life situation of HCW impacts the outcome of a PCC indication. Studies reported a 50% higher risk for nurses with two or more of the following preinfectional indications for depression and anxiety: Loneliness, perceived stress, depression, anxiety and worry about COVID (Wang et al. 2022).

Milde et al. (2023) found in a small sample–sized study that psychological factors predict the severity and number of PCC symptoms. They found stress, depression and fear of COVID‐19 health consequences to increase the symptom burden. PCC patients with high levels of depression and fatigue tended to rate all facets of their health worse (Ruzicka et al. 2023). The same survey showed substantial discrepancies in the perception of illness severity between PCC patients and their attending physicians. Perception of PCC illness severity seemed to be prone to bias (Ruzicka et al. 2023). Thomason et al. (2022) showed that perceived socioeconomic status (SES) and discrimination increased lasting cognitive symptoms for long‐COVID. However, objective SES was nonsignificant as a predictor. However, results are diverse, and SES has been found to be associated with PCC in other settings (Morrow et al. 2024).

## 3 Aims

Mental health has been found to predict PCC symptom load, but only in small samples, foreign studies or studies outside a health care setting (Milde et al. 2023; Thomason et al. 2022). Hence, in the present study, mental health factors and social factors are included in a large cohort of German HCW and can give a more complex insight into PCC.

The aims of this cross‐sectional study are as follows:

1. To identify HCW with PCC in the study sample and examine whether they differ in their post‐COVID typical symptomology from HCW without PCC.
2. To assess whether HCW with PCC experience higher levels of depression, anxiety and posttraumatic symptoms, more work–family conflict, less social support, less sense of coherence and worse working conditions than HCW without PCC.
3. To investigate, if those factors are associated with the perceived severity of PCC symptoms and can be identified as risk or protective factors.

## 4 Methods

### 4.1 Designs and Settings

The study design was cross‐sectional and observational. Data were collected between April and June 2023 by the psychosomatic departments of the University Hospitals of Erlangen, Bonn, Cologne, Dresden and Ulm. Further hospitals and networks supported and promoted the study. The survey lasted approximately 20–30 min. The survey language was German and consisted of 127 items. The translations were proofread and reviewed by the coauthors to ensure that no information was conveyed incorrectly.

### 4.2 Participants and Data Collection

Participants with a minimum age of 18 years working in the German healthcare sector, predominantly from hospitals, were included. In total, 2768 HCW were included who came from various fields of medical service and age groups and were mostly female. The link to the study was shared via mailing lists or online platforms. The study used the Unipark (www.unipark.com) and SoSci Survey (www.soscisurvey.com) tool. Our data emerge from the VOICE online self‐report survey addressing HCW in Germany. The VOICE survey is part of egePAN Unimed, researching COVID‐19, and was partially funded by the German Federal Ministry of Education and Research. This paper contains only data from the fifth wave of the survey.

### 4.3 Ethical Considerations

The study was approved by the Ethics Committee of the Medical Faculty of the Friedrich‐Alexander University Erlangen‐Nürnberg (FAU), according to the Declaration of Helsinki (reference number 20‐133‐B).

### 4.4 Measurements

Included sociodemographic sample characteristics were occupation, gender, age group, migration background, employment status, direct/indirect patient care and work experience.

For this study, PCC was assessed via self‐report by the item ‘Have you already been infected with SarS‐CoV‐2?’ Participants could respond ‘Yes, with persistent symptoms or long‐lasting health effects (longer than 3 months)’, indicating PCC according to the WHO definition. Other possible responses were ‘Yes, without symptoms’, ‘Yes only with acute symptoms (COVID‐19)’ and ‘No/ I don't know’.

Further, PCC severity measured by a self‐developed screening instrument asking ‘How much did you suffer in the last seven days from…’ with the possible responses: ‘feeling of faintness and dizziness’, ‘heart and chest pain’, ‘nausea or indigestion’, ‘weakness in different parts of the body’, ‘difficulty breathing’, ‘sleeping problems’, ‘physical or psychological exhaustion’, ‘concentration or memory problems’, ‘headache’ and ‘loss of smell or taste’. Items were answered on a scale from 1 (not at all) to 5 (very strong). The post‐COVID sum score (PCSS) was calculated from the item scores. The chosen items were derived from different relevant meta‐analyses on PCC symptoms (Davis et al. 2021; Han et al. 2022; Premraj et al. 2022). The internal consistency of PCCS was Cronbach's *α* = 0.853 and deemed acceptable.

Working conditions were measured by a self‐developed screening instrument: Working condition sum score (WCSS), assessed with five items scaled from 0 (completely disagree) to 4 (completely agree) and means were computed with the following items: ‘I work more than before the COVID‐19 pandemic’ (inverted); ‘I feel sufficiently informed about COVID‐19’; ‘There is sufficient staff for the current workload’; ‘I can recover sufficiently during my free time’ and ‘I can trust in my colleagues when it gets difficult during work’. Higher sum scores reflect more comfortable working conditions, while lower scores reflect less comfortable ones. Internal consistency with Cronbach's *α* = 0.729 was acceptable.

The German version of the Patient Health Questionnaire 4 (PHQ‐4) was applied to measure depression and anxiety (Löwe et al. 2010). The PHQ‐4 consists of four items, two forming the Patient Health Questionnaire 2 (PHQ‐2) for depression and the other two forming the Generalised Anxiety Disorder 2 (GAD‐2) for anxiety respectively. The sum score for each test ranges from 0 (not at all) to 6 (nearly every day); a cut‐off value of ≥ 3 is used to identify clinically significant levels for PHQ‐2 and GAD‐2, comprising only two items. Spearman–Brown coefficients were calculated with internal consistencies of *ρ* = 0.778 and *ρ* = 0.812 respectively (Eisinga, Grotenhuis, and Pelzer 2013).

The Impact of Event Scale (IES‐6) was used to measure PTSD (post‐traumatic stress disorder) symptoms (Horowitz, Wilner, and Alvarez 1979; Thoresen et al. 2010). The scale consists of six items ranging from 0 to 30, each ranging from 0 (not at all) to 4 (often). The six items capture the three PTSD symptom clusters: intrusion, avoidance/numbing and hyperarousal (Thoresen et al. 2010). The participants were asked to answer the items considering COVID‐19. For the current sample, Cronbach's *α* = 0.731 was computed.

Work–family conflicts were measured by the Work–Family Conflict Scale (WFC) and the Family‐Work Conflict Scale (FWC), originally consisting of each five items (Netemeyer, Boles, and McMurrian 1996). Only four items were employed in the applied version. Items were scaled from 0 (not at all) to 5 (yes, exactly). The samples' internal consistency was calculated with Cronbach's *α* = 0.864.

The German version of the ENRICHD Social Support Inventory (ESSI) assesses emotional social support (Kendel et al. 2011). Five items are summed up to a score from 5 to 25, scaled from 1 (none of the time) to 5 (all of the time). Higher values intend higher social support. Critical low values are indicated by scores of ≤ 18 and at least two items ≤ 3 (The ENRICHD Investigators 2000). ESSI's internal consistency for this sample was computed with Cronbach's *α* = 0.911.

The German adaptation of the Sense of Coherence‐3 Scale (SOC‐3), initially developed by Antonovsky (Antonovsky 1993), measured sense of coherence. The SOC‐3 is an ultrashort questionnaire consisting of three items scaled from 0 (very often) to 7 (very seldom or never). The third item is inverted 0 (‘feel how good it is to be alive’) to 7 (‘ask yourself why you exist at all’). Sense of coherence is a psychological resource that leads to resilience towards stressors (Antonovsky 1993). It measures people's perception of how comprehensible, manageable and meaningful their world is (Antonovsky 1993). Cronbach's *α* = 0.725 was acceptable.

### 4.5 Data Analysis

Data analyses were performed with IBM SPSS Version 29. Absolute frequencies and percentages were calculated to describe sociodemographic sample characteristics and PCC indication. Group differences between recipients with self‐reported PCC and no PCC for PHQ‐2, GAD‐2, IES‐6, WFC, ESSI, SOC‐3 and WCSS were calculated with two‐tailed *t*‐tests for independent samples. Because of the *p*‐values' robustness below *p* < 0.001, *α*‐error adjustment was unrequired. Effect sizes were reported with Cohen's *d*: Small = *d* ≥ 0.2, medium = *d* ≥ 0.5 and large = *d* ≥ 0.8 (Cohen 1988). Due to unequal variances, Welch's test was chosen to measure group differences (Rasch and Guiard 2004). A linear regression was calculated for people with self‐reported PCC indication to investigate the influence on the PCSS (dependent variable). A forced entry was chosen, and variance inflation factors (VIF) were reported. VIF below 10 is deemed acceptable. The following independent variables were included: Age group, gender, migration background, occupation, PHQ‐2, GAD‐2, IES‐6, WFC, ESSI, SOC‐3 and WCSS. The level of significance was set to *p* < 0.05 for all calculations.

## 5 Results

### 5.1 Characteristics of the Sample

Table 1 shows the sociodemographic characteristics, including occupation, gender, age, migration background, employment status, direct patient care, work experience and prevalence of PCC.

**TABLE 1:** Sociodemographic characteristics among HCW (*N* = 2768).

	Total sample	PCC	Non‐PCC
	*N*	*n*	*n*
PCC	332 (12.0%)	—	—
Occupation			
Physicians	364 (13.1%)	27 (7.4%)	337 (92.6%)
Nurses	775 (28.0%)	120 (15.5%)	655 (84.5%)
Medical technical assistants (MTA)	375 (13.5%)	48 (12.8%)	327 (87.2%)
Psychologists/psychotherapists	85 (3.1%)	9 (10.6%)	76 (89.4%)
Health therapists	97 (3.5%)	7 (7.2%)	90 (92.8%)
Students/trainees/study nurses	110 (4.0%)	14 (12.7%)	96 (87.3%)
Research assistant	138 (5.0%)	11 (8.0%)	127 (92.0%)
Administrative staff	353 (12.8%)	42 (11.9%)	311 (88.1%)
Other	472 (17.0%)	37 (7.8%)	435 (92.2%)
Gender			
Men	685 (24.7%)	49 (7.2%)	636 (92.8%)
Women	2075 (75.0%)	280 (13.5%)	1795 (86.5%)
Gender diverse	8 (0.3%)	3 (37.5%)	5 (62.5%)
Age group			
18–30 years	539 (19.5%)	73 (13.5%)	466 (86.5%)
31–40 years	741 (26.8%)	75 (10.1%)	666 (89.9%)
41–50 years	593 (21.4%)	70 (11.8%)	523 (88.2%)
51–60 years	695 (25.1%)	94 (13.5%)	601 (86.5%)
Over 60 years	200 (7.2%)	20 (10.0%)	180 (90.0%)
Migration background*			
No	2441 (87.1%)	345 (11.6%)	2096 (88.4%)
Yes	327 (12.9%)	58 (17.7%)	269 (82.3%)
Employment status			
Full‐time	1716 (62.0%)	205 (12.0%)	1511 (88.0%)
Part‐time	1052 (38.0%)	127 (12.1%)	925 (87.9%)
Direct patient care			
Yes	1706 (61.6%)	212 (12.4%)	1494 (87.6%)
No	1062 (38.4%)	120 (11.3%)	925 (88.7%)
Work experience			
< 3 years	142 (8.3%)	15 (10.6%)	127 (89.4%)
3–6 years	223 (13.0%)	34 (15.2%)	189 (84.8%)
> 6 years	1346 (79.7%)	163 (12.1%)	1183 (87.9%)

Abbreviation: PCC, post‐COVID condition.

* Migration background means that the participant or at least one of the participant's parents did not have German citizenship by birth. Percentages refer to the total group and PCC subgroup respectively.

Of the 2768 participants, 12.0% (*n* = 332) indicated PCC according to the WHO criterion. The most frequent occupation in the entire group was nurses (28%, *n* = 775), followed by medical technical assistants (13.5%, *n* = 375), physicians (13.1%, *n* = 363), administrative staff (12.8%, *n* = 353), research assistants (5.0%, *n* = 138), students/trainees/study nurses (4.0%, *n* = 110), health therapists, consisting of physiotherapists, occupational therapists, music therapists, speech therapists (3.5%, *n* = 97) and psychologists/psychotherapists (3.1%, *n* = 85). Other occupations were combined as ‘other’ with 17.0% (*n* = 472).

Three of four (75.0%, *n* = 2075) participants were female, and *n* = 8 (0.3%) stated to have a diverse gender. Due to their low number, for statistical reasons, gender‐diverse respondents were not considered for analyses. The age groups were 18–30, 19.5% (*n* = 539); 31–40, 26.8% (*n* = 741); 41–50, 21.4% (*n* = 593) and 51–60 years; 25.1% (*n* = 695). The smallest group were HCW over 60 years with 7.2% (*n* = 200). The sample consisted mainly of German Natives without migration background with 87.1% (*n* = 2441). Of the sample, 62.0% (*n* = 1716) worked full‐time, and 61.6% (*n* = 1706) were deployed in direct patient care. For 79.7% (*n* = 1346), the work experience was above 6 years, while 8.3% (*n* = 142) had less than 3 years of work experience, and 13.0% (*n* = 223) had 3–6 years.

### 5.2 Group Differences Between HCW With PCC and HCW Without PCC for Specific PCC Symptoms

Table 2 shows differences in PCC symptoms between groups classified based on the WHO criterion. For all tests, the PCC group showed higher scores. The differences between the groups were significant for all PCC symptoms with *p* < 0.001 and Cohen's *d* effect sizes ranged between 0.517 and 0.885. A strong effect was found for PCSS with *d* = 1.024. Other large effects were found for concentration or memory problems (*d* = 0.804) and difficulty breathing (*d* = 0.885). Eight effects were medium, ranging from *d* = 0.517 (feeling of faintness and dizziness) to *d* = 0.775 (weakness in different parts of the body).

**TABLE 2:** Group differences between HCW with and without PCC for PCC symptoms.

	HCW with PCC			HCW without PCC			*t*	*p*	Cohen‘s *d*
	*n*	*M*	SD	*n*	*M*	SD			
PCSS	296	22.84	7.79	2123	16.72	5.68	−13.044	< 0.001	1.024
Difficulty breathing	296	2.04	1.16	2112	1.36	0.71	−9.941	< 0.001	0.885
Concentration or memory problems	295	2.88	1.29	2114	2.00	1.07	−11.176	< 0.001	0.804
Weakness in different body parts	296	2.21	1.27	2111	1.50	0.86	−9.364	< 0.001	0.775
Loss of smell or taste	296	1.53	1.08	2119	1.10	0.46	−6.764	< 0.001	0.751
Physical or psychological exhaustion	296	3.20	1.35	2115	2.42	1.15	−9.556	< 0.001	0.670
Heart and chest pain	295	1.66	0.96	2119	1.26	0.61	−6.920	< 0.001	0.599
Nausea or indigestion	296	2.18	1.21	2115	1.64	0.91	−7.503	< 0.001	0.573
Sleeping problems	296	2.96	1.36	2117	2.26	1.20	−8.335	< 0.001	0.569
Headache	296	2.53	1.29	2116	1.94	1.07	−7.476	< 0.001	0.535
Feeling of faintness and dizziness	294	1.68	1.00	2121	1.31	0.67	−6.134	< 0.001	0.517

*Note:* Varying sample sizes due to missing values.

Abbreviations: HCW, healthcare workers; PCC, post‐COVID condition; PCSS, Post‐COVID Symptom Sum Score.

### 5.3 Group Differences for Measures of Depression, Anxiety and PTSD Symptoms, Work–Family Conflict, Social Support, Sense of Coherence, Working Conditions

Table 3 illustrates group differences across different scales. All tests were significant *p* < 0.001, and effect sizes of Cohen's *d* ranged between 0.254 and 0.629. Significantly higher levels of depressive symptoms, anxiety symptoms, PTSD symptoms and work–family conflict were found for the PCC group (*p*'s < 0.001). HCW without PCC scored significantly higher for perceived working conditions, social support, sense of coherence (WCSS, ESSI and SOC‐3) (*p*'s < 0.001). Small effects were found for WFC (*d* = 0.291), ESSI (*d* = 0.351), SOC‐3 (*d* = 0.254), WCSS (*d* = 0.441) and medium effect sizes for PHQ‐2 (*d* = 0.624) and GAD‐2 (*d* = 0.629) and for IES‐6 (*d* = 0.552).

**TABLE 3:** Group differences between HCW with PCC and without PCC for PHQ‐2, GAD‐2, IES‐6, WFC, ESSI, SOC and WCSS.

	HCW with PCC			HCW without PCC			*t*	*p*	Cohen‘s *d*
	*N*	*M*	SD	*n*	*M*	SD			
PHQ‐2 (depressive symptoms)	298	2.53	1.889	2163	1.60	1.42	−8.183	< 0.001	0.624
GAD‐2 (anxiety symptoms)	298	2.38	1.871	2163	1.41	1.48	−8.547	< 0.001	0.629
IES‐6 (PTSD symptoms)	299	2.51	0.698	2173	2.15	0.69	−8.340	< 0.001	0.552
WFC (work–family conflict)	312	2.76	1.028	2264	2.47	0.98	−4.662	< 0.001	0.291
ESSI (social support)	298	3.92	0.964	2150	4.21	0.81	−8.340	< 0.001	0.351
SOC‐3 (sense of coherence)	296	4.10	0.959	2138	4.31	0.84	3.693	< 0.001	0.254
WCSS (working conditions)	328	3.03	0.714	2406	3.33	0.68	−12.892	< 0.001	0.441

*Note:* For PHQ‐2, GAD‐2, IES‐6 and WFC, higher values indicate severe symptoms, while for ESSI, SOC and WCSS, higher values indicate stronger social support, sense of coherence and better working conditions.

Abbreviations: ESSI, ENRICHD Social Support Inventory; GAD‐2, Generalised Anxiety Disorder‐2; IES‐6, Impact of Event Scale‐6; PHQ‐2, Patient Health Questionnaire‐2; SOC‐3, Sense Of Coherence‐3 Scale; WCSS, Working Condition Sum Score; WFC, Work–Family Conflict Scale; HCW, healthcare workers; PCC, post‐COVID condition; PTSD, post‐traumatic stress disorder.

### 5.4 Risk and Protective Factors for PCSS

Table 4 shows the results of the linear regression analysis investigating the association between investigated variables and PCSS for HCW with PCC. The final regression model explained 50.7% (*R* ^2^_adjusted_) of the variance. For sociodemographic characteristics, only migration background (*p* = 0.002, *β* = 0.133) was a significant predictor for higher PCSS. Only the occupational group physicians (ref. = nurses) were a significant predictor for lower PCSS (*p* = 0.002, *β* = −146). The WCSS did not show any significance (*p* = 0.904). PCSS was associated with more depressive symptoms (*p* = 0.001, *β* = 0.248), more anxiety symptoms (*p* = < 0.001, *β* = 0.223), more PTSD symptoms (*p* = 0.016, *β* = 0.122) and more work–family conflict (*p* < 0.001, *β* = 0.2). Less perceived social support (*p* = 0.015, *β* = −0.109) and sense of coherence (*p* = 0.003, *β* = −0.135) predicted higher PCSS values.

**TABLE 4:** Linear regression analysis for severity of PCCS for HCW with PCC (*N* = 296).

Independent variables	PCSS					
	Regression coefficient (CI 95%)	SE	Beta	*t*	VIF	*p*
Constant	13.402 (6.179, 20.626)	3.669		3.653		**< 0.001**
Age group	0.058 (−0.510, 0.626)	0.289	0.009	0.202	1.292	0.775
Gender	0.768 (−1.107, 2.643)	0.952	0.035	0.806	1.082	0.421
Migration background*	3.060 (1.175, 4.945)	0.957	0.138	3.196	1.091	**0.002**
Occupation						
Nurses	Ref.					
Physicians	−4.002 (−6.727, −1.2779)	1.384	−0.146	−2.891	1.097	**0.004**
Medical technical assistants (MTA)	−0.003 (−2.629, 6.976)	1.257	< 0.001	−0.002	1.160	0.563
Psychologists/psychotherapists	−1.781 (−6.023, 2.461)	2.155	−0.037	−0.826	1.070	0.472
Health therapists	2.173 (−2.629, 6.976)	2.440	0.039	0.891	1.054	0.232
Students/trainees/study nurses	−0.533 (−4.336, 3.269)	1.932	−0.013	−0.276	1.061	0.880
Research assistants	−0.367 (−4.404, 3.670)	2.051	−0.008	−0.179	1.127	0.969
Administrative staff	1.080 (−1.345, 3.504)	1.231	0.049	0.877	1.229	0.126
PHQ‐2 (depressive symptoms)	0.981 (0.437, 1.524)	0.276	0.238	3.553	2.655	**0.001**
GAD‐2 (anxiety symptoms)	0.943 (0.392, 1.495)	0.280	0.228	3.366	2.695	**< 0.001**
IES‐6 (PTSD symptoms)	1.353 (0.258, 2.448)	0.556	0.120	2.433	1.407	**0.016**
WFC (work–family conflict)	1.535 (0.751, 2319)	0.398	0.200	3.853	1.591	**< 0.001**
ESSI (social support)	−0.882 (−1.589, −0.175)	0.359	−0.109	−2.456	1.152	**0.015**
SOC‐3 (sense of coherence)	−1.109 (−1.862, 0.766)	0.372	−0.135	−2.978	1.217	**0.003**
WCSS (working conditions)	0.202 (−0.362, 0.766)	0.286	0.033	0.706	1.326	0.904

*Note:* Gender excludes gender‐diverse people due to the low sample size. Age groups are one = 18–30 years, 2 = 31–40 years, 3 = 41–50 years, 4 = 51–60 years, 5 = over 60 years. *R* ^2^ = 0.511. *R* ^2^_adjusted_ = 0.507. For PHQ‐2, GAD‐2, IES‐6, WFC higher values indicate severe symptoms, while for ESSI, SOC, WCSS higher values indicate stronger social support, sense of coherence and better working conditions. Bold values are significant for *p* < 0.05.

Abbreviations: ESSI, ENRICHD Social Support Inventory; GAD‐2, Generalised Anxiety Disorder‐2; IES‐6, Impact of Event Scale‐6; PCSS, Post‐COVID Symptom Sum Score; PHQ‐2, Patient Health Questionnaire‐2; SOC‐3, Sense Of Coherence‐3 Scale; WCSS, Working Condition Sum Score; WFC, Work–Family Conflict Scale. HCW, healthcare workers; PCC, post‐COVID condition.

* Migration background means that the participant or at least one of the participant's parents did not have German citizenship by birth.

## 6 Discussion

This study assessed HCW who fulfil the criteria of PCC and examined the differences between HCW with and without reported PCC. General health, mental health, resources and working conditions were analysed. The primary aim was to point out the potential risk and protective factors associated with the severity of PCC symptoms in a large sample of HCW. Primary conclusions are: (1) HCW who reported PCC showed severe PCC symptoms, (2) the PCC group's mental health was more affected than HCW without PCC, (3) small differences showed for social support, sense of coherence, work–family conflict and working condition, (4) sociodemographic factors' (physician, migration background) association with lower and, respectively, higher PCSS values point to socioeconomic status playing a role in reporting PCC symptoms. (5) depression, anxiety and PTSD symptoms were associated with reporting severe PCC symptoms and are in line with previous results from other fields.

HCW with a self‐reported PCC condition showed significantly severe symptoms, which are often described in PCC, compared to HCW who did not report a PCC condition. Ruzicka et al. (2023) reported discrepancies between the self‐perception of PCC symptoms and diagnoses of attending medical staff. Considering that our study is based on in‐work HCW, excluding PCC cases unable to work, and the self‐reporting nature of the VOICE study, potential overreporting for PCC symptoms or assessed psychological variables need to be regarded. The above‐mentioned working group (2023) explained dissonant disease perception by the sample's young medium age of 39 years and the recipients' missing experience with severe diseases, which may shape their health perception. The PCC subgroup of our study ranges between the age groups of 31–40 and 41–50 years on average and might be similar to the age group in Ruzicka et al. (2023). However, all study participants work in the healthcare sector and might have an enhanced understanding of disease severity. Otherwise, the PCC subgroup may be in a severely worse health state.

HCW with PCC reported more psychological distress, and effect sizes for PHQ‐2, GAD‐2 and IES‐6 were medium. In contrast, the effect sizes for social support (ESSI), sense of coherence (SOC‐3), work–family conflict (WFC) and working conditions (WCS) were small. The results of HCW with PCC being impaired with depression and anxiety are in line with study results from other studies investigating the same topic for different populations (Cacciatore et al. 2022; Frontera et al. 2021; Hasenoehrl et al. 2023; Houben‐Wilke et al. 2022; Matsumoto et al. 2022; Morrow et al. 2024; Ruzicka et al. 2023; Steinmetz et al. 2023; Tebeka et al. 2023). Trauma is less often investigated in post‐COVID/long‐COVID samples, but associations between trauma symptoms and post‐COVID/long‐COVID are commonly found in different populations (Craparo et al. 2022; Houben‐Wilke et al. 2022; Matsumoto et al. 2022; Steinmetz et al. 2023). Effect sizes for ESSI, WFC, SOC and WCS showed that HCW with PCC rated their work environment and the support they received on the job lower than those without symptoms. Problems between their work and family environment appear more often for HCW with PCC. It can be assumed that, in consequence, resources are reduced for HCW with PCC. Shi et al. (2021) assessed the relationship between work stress, social support, resilience, depression and anxiety for Chinese HCW during the pandemic. They found social support and resilience to be mediators between work stress and depression/anxiety. An earlier report from the VOICE study found similar SOC results but no correlations between mental health and social support for physicians and MTAs (Schmuck et al. 2021). Another VOICE article researched working conditions and mental health for HCW during the pandemic: worse‐rated working conditions were associated with lower mental health (Borusiak et al. 2022). Lower mental health scores and resources for HCW with PCC simultaneously raise the question about the relationship between these factors. Lower available resources may increase the impact of PCC symptomology on mental health. It is also conceivable that PCC stresses resources to the point that they cannot shield mental health.

In contrast to previous research, the regression results for age group and gender revealed no association with PCC severity. Our study was composed of three of four women, which is in line with the proportion of women in health care in Europe (World Health Organisation 2022). A migration background was a significant factor for higher PCSS values, while being a physician (in ref. to nurses) was a significant predictor for lower PCSS values in our regression model. Both can be interpreted as markers of socioeconomic status (Groene et al. 2023; Tillmann et al. 2018). In a Scottish sample, recipients from socioeconomically deprived areas had more severe, long‐lasting COVID symptoms (Morrow et al. 2024). The authors pointed out that those recipients were more likely to interact with unhealthy behaviour. Thomason et al. (2022) found similar effects on socioeconomic status associated with PCC severity, but the effects were found on perceived socioeconomic status and discrimination. No results were found for objective socioeconomic status. The ranking of one's social position could contribute to reporting more severe PCC symptoms.

The regression model revealed that HCW with PCC, who report more PTSD, depression and anxiety symptoms, score higher for PCSS. Another study found that psychological factors are not side effects or disease consequences and influence COVID‐19 symptoms (Milde et al. 2023). Engelmann et al. (2022) also found mental burden associated with somatic symptoms to be a predictor for deteriorating PCC symptoms. WCS were insignificant in our model, which shows that a higher rating of PCSS was not associated with a generally worse perception of participants' work environment. Several authors favour the biopsychosocial model for investigating long‐/post‐COVID (Bisenius and Kersting 2022; Engel 1978; Engelmann et al. 2023; Thurner and Stengel 2023). The biopsychosocial model could also explain why lower resources (in our study: ESSI, SOC) and existing work–family conflicts (WFC) were associated with reporting more severe PCC symptoms. It also could explain the association between higher PCSS values and higher mental load (in our study: PHQ‐2, GAD‐2, IES‐6). Bisenius and Kersting (2022) have adapted the biopsychosocial model for prolonged COVID‐19: Social, psychological and biological factors interact before infection and collectively influence the initial course of COVID‐19 and, separately, the development of psychological and physical symptoms (Bisenius and Kersting 2022; Thurner and Stengel 2023). The progression of the disease and the physical and psychological/social consequences also mutually and jointly affect the development of physical, psychological and social long‐term consequences of COVID‐19 (Bisenius and Kersting 2022; Thurner and Stengel 2023). Missing resources can interact during that process before infection and increase the long‐term consequences of the disease (Bisenius and Kersting 2022; Thurner and Stengel 2023). The biopsychosocial model could explain why lower resources (in our study: ESSI, SOC) and existing work–family conflicts (WFC) were associated with reporting more severe PCC symptoms. It also could explain the association between higher PCSS values and higher mental load (in our study: PHQ‐2, GAD‐2, IES‐6).

In the same regression model for the non‐PCC group (data not provided), more demographic variables were statistically significant: Female gender, migration background, occupational groups of physicians, MTA and students, as well as high values for PHQ‐2, GAD‐2, IES‐6, WFC and WCS and lower values for ESSI, and SOC all were significantly associated with higher symptom scores. More significant independent variables show that the underlying conditional framework is more comprehensive for the non‐PCC group, which means more factors must be involved in the development of the syndrome. Conversely, there is more scope for coping with the disease via the reported variable factors.

### 6.1 Strengths and Limitation

A strength of the study is the large sample size, which increases its statistical power and makes it more likely to detect small effects. Several psychological stress and resilience factors were examined, allowing us to investigate the relationship between many aspects and a conditional framework in one sample.

The present study has some limitations. Due to the study sample, our results are restricted to HCW at work, excluding people on sick leave, who might have more severe symptoms of PCC. This study group is not representative of the general population. The study design is cross‐sectional, and causal conclusions are not possible. The PCC indication is self‐reported and not diagnosed by medical personnel, decreasing reliability. Leading symptoms of the disease, such as fatigue and postexertional malaise, were not explicitly investigated because they were not described specifically at this early stage. The study was designed and executed in German, which could have excluded people with poor German language skills.

## 7 Conclusion

The study found that psychological factors should be considered in PCC research. It demonstrated that individuals who considered themselves to be ill with PCC differed significantly in both their physical and psychological condition. However, it is important to note that the definition of PCC and its symptoms is broad. Future research needs to continue focusing on biomarkers or other distinct features for reliable identification of PCC. It will be beneficial to research biopsychosocial interactions and illness perceptions in PCC patients to deepen the understanding of those processes in general. To elaborate on the biopsychosocial theory, research on existing data should focus on the association between factors associated with mental burden and PCC ratings to understand causal and temporal interaction. The fact that individuals with this type of diagnosis worry about stigmatisation will make medical and psychological help more complex.

## Conflicts of Interest

The authors declare no conflicts of interest.

## Supporting information


Data S1.

## Data Availability

The data that support the findings of this study are available from the corresponding author upon reasonable request.
