# Supplementary material for: Psychological and Social Factors Associated With Reporting Post‐COVID Symptoms Among German Healthcare Workers: A Cross‐Sectional Study
Source: J Clin Nurs. 2025 Jan 14;34(10):4095–104. doi: 10.1111/jocn.17608 (PMC12409199; doi:10.1111/jocn.17608)
Supplement: Supplementary file 1 — Data S1. [file JOCN-34-4095-s001.pdf]

APA Style JARS
Journal Article Reporting Standards

JARS–**Quant** | Table 1
Information Recommended for Inclusion in Manuscripts
That Report New Data Collections Regardless of Research Design

Title and Title Page

Title

- Identify main variables and theoretical issues under investigation and the relationships between them.
- Identify the populations studied.

Author Note

- Provide acknowledgment and explanation of any special circumstances, including
  - registration information if the study has been registered
  - use of data also appearing in previous publications
  - prior reporting of the fundamental data in dissertations or conference papers
  - sources of funding or other support
  - relationships or affiliations that may be perceived as conflicts of interest
  - previous (or current) affiliation of authors if different from location where the study was conducted
  - contact information for the corresponding author
  - additional information of importance to the reader that may not be appropriately included in other sections of the paper

Abstract

Objectives

- State the problem under investigation, including main hypotheses.

Participants

- Describe subjects (nonhuman animal research) or participants (human research), specifying their pertinent characteristics for the study; in animal research, include genus and species. Participants are described in greater detail in the body of the paper.

Study Method

- Describe the study method, including
  - research design (e.g., experiment, observational study)
  - sample size
  - materials used (e.g., instruments, apparatus)
  - outcome measures
  - data-gathering procedures, including a brief description of the source of any secondary data. If the study is a secondary data analysis, so indicate.

Findings

- Report findings, including effect sizes and confidence intervals or statistical significance levels.

Conclusions

- State conclusions, beyond just results, and report the implications or applications.

Introduction

Problem

- State the importance of the problem, including theoretical or practical implications.

Review of Relevant Scholarship

- Provide a succinct review of relevant scholarship, including
  - relation to previous work
  - differences between the current report and earlier reports if some aspects of this study have been reported on previously

Hypothesis, Aims, and Objectives

- State specific hypotheses, aims, and objectives, including
  - theories or other means used to derive hypotheses
  - primary and secondary hypotheses
  - other planned analyses
- State how hypotheses and research design relate to one another.

Method

Inclusion and Exclusion

- Report inclusion and exclusion criteria, including any restrictions based on demographic characteristics.

Participant Characteristics

- Report major demographic characteristics (e.g., age, sex, ethnicity, socioeconomic status) and important topic-specific characteristics (e.g., achievement level in studies of educational interventions).
- In the case of animal research, report the genus, species, and strain number or other specific identification, such as the name and location of the supplier and the stock designation. Give the number of animals and the animals’ sex, age, weight, physiological condition, genetic modification status, genotype, health–immune status, drug or test naïveté, and previous procedures to which the animal may have been subjected.

© 2020 American Psychological Association

Sampling Procedures

- Describe procedures for selecting participants, including
  - sampling method if a systematic sampling plan was implemented
  - percentage of sample approached that actually participated
  - whether self-selection into the study occurred (either by individuals or by units, such as schools or clinics)
- Describe settings and locations where data were collected as well as dates of data collection.
- Describe agreements and payments made to participants.
- Describe institutional review board agreements, ethical standards met, and safety monitoring.

Sample Size, Power, and Precision

- Describe the sample size, power, and precision, including
  - intended sample size
  - achieved sample size, if different from the intended sample size
  - determination of sample size, including
    - › power analysis, or methods used to determine precision of parameter estimates
    - › explanation of any interim analyses and stopping rules employed

Measures and Covariates

- Define all primary and secondary measures and covariates, including measures collected but not included in the report.

Data Collection

- Describe methods used to collect data.

Quality of Measurements

- Describe methods used to enhance the quality of measurements, including
  - training and reliability of data collectors
  - use of multiple observations

Instrumentation

- Provide information on validated or ad hoc instruments created for individual studies, for individual studies (e.g., psychometric and biometric properties).

Masking

- Report whether participants, those administering the experimental manipulations, and those assessing the outcomes were aware of condition assignments.
- If masking took place, provide a statement regarding how it was accomplished and whether and how the success of masking was evaluated.

Psychometrics

- Estimate and report values of reliability coefficients for the scores analyzed (i.e., the researcher's sample), if possible. Provide estimates of convergent and discriminant validity where relevant.
- Report estimates related to the reliability of measures, including
  - interrater reliability for subjectively scored measures and ratings
  - test–retest coefficients in longitudinal studies in which the retest interval corresponds to the measurement schedule used in the study
  - internal consistency coefficients for composite scales in which these indices are appropriate for understanding the nature of the instruments being used in the study
- Report the basic demographic characteristics of other samples if reporting reliability or validity coefficients from those samples, such as those described in test manuals or in norming information for the instrument.

Conditions and Design

- State whether conditions were manipulated or naturally observed. Report the type of design as per the JARS–Quant tables:
  - experimental manipulation with participants randomized
    - › Table 2 and Module A
  - experimental manipulation without randomization
    - › Table 2 and Module B
  - clinical trial with randomization
    - › Table 2 and Modules A and C
  - clinical trial without randomization
    - › Table 2 and Modules B and C
  - nonexperimental design (i.e., no experimental manipulation): observational design, epidemiological design, natural history, and so forth (single-group designs or multiple-group comparisons)
    - › Table 3
  - longitudinal design
    - › Table 4
  - *N*-of-1 studies
    - › Table 5
  - replications
    - › Table 6
- Report the common name given to designs not currently covered in JARS–Quant.

Data Diagnostics

- Describe planned data diagnostics, including
  - criteria for post-data-collection exclusion of participants, if any
  - criteria for deciding when to infer missing data and methods used for imputation of missing data
  - definition and processing of statistical outliers
  - analyses of data distributions
  - data transformations to be used, if any

Analytic Strategy

- Describe the analytic strategy for inferential statistics and protection against experiment-wise error for
  - primary hypotheses
  - secondary hypotheses
  - exploratory hypotheses

Results

Participant Flow

- Report the flow of participants, including
  - total number of participants in each group at each stage of the study
  - flow of participants through each stage of the study (include figure depicting flow, when possible; see the JARS–Quant Participant Flowchart)

Recruitment

- Provide dates defining the periods of recruitment and repeated measures or follow-up.

Statistics and Data Analysis

- Provide information detailing the statistical and data-analytic methods used, including
  - missing data
    - frequency or percentages of missing data
    - empirical evidence and/or theoretical arguments for the causes of data that are missing—for example, missing completely at random (MCAR), missing at random (MAR), or missing not at random (MNAR)
    - methods actually used for addressing missing data, if any
  - descriptions of each primary and secondary outcome, including the total sample and each subgroup, that includes the number of cases, cell means, standard deviations, and other measures that characterize the data used
  - inferential statistics, including
    - results of all inferential tests conducted, including exact *p* values if null hypothesis significance testing (NHST) methods were used, and reporting the minimally sufficient set of statistics (e.g., *df*s, mean square [*MS*] effect, *MS* error) needed to construct the tests
    - effect-size estimates and confidence intervals on estimates that correspond to each inferential test conducted, when possible
    - clear differentiation between primary hypotheses and their tests–estimates, secondary hypotheses and their tests–estimates, and exploratory hypotheses and their test–estimates

Statistics and Data Analysis *(continued)*

  - complex data analyses—for example, structural equation modeling analyses (see also Table 7), hierarchical linear models, factor analysis, multivariate analyses, and so forth, including
    - details of the models estimated
    - associated variance–covariance (or correlation) matrix or matrices
    - identification of the statistical software used to run the analyses (e.g., SAS PROC GLM or the particular R package)
  - estimation problems (e.g., failure to converge, bad solution spaces), regression diagnostics, or analytic anomalies that were detected and solutions to those problems.
  - other data analyses performed, including adjusted analyses, if performed, indicating those that were planned and those that were not planned (though not necessarily in the level of detail of primary analyses).
- Report any problems with statistical assumptions and/or data distributions that could affect the validity of findings.

Discussion

Support of Original Hypotheses

- Provide a statement of support or nonsupport for all hypotheses, whether primary or secondary, including
  - distinction by primary and secondary hypotheses
  - discussion of the implications of exploratory analyses in terms of both substantive findings and error rates that may be uncontrolled

Similarity of Results

- Discuss similarities and differences between reported results and work of others.

Interpretation

- Provide an interpretation of the results, taking into account
  - sources of potential bias and threats to internal and statistical validity
  - imprecision of measurement protocols
  - overall number of tests or overlap among tests
  - adequacy of sample sizes and sampling validity

Generalizability

- Discuss generalizability (external validity) of the findings, taking into account
  - target population (sampling validity)
  - other contextual issues (setting, measurement, time; ecological validity)

Implications

- Discuss implications for future research, program, or policy.

© 2020 American Psychological Association
